# Elevated YKL‐40 serum levels may contribute to wet age‐related macular degeneration via the ERK1/2 pathway

**DOI:** 10.1002/2211-5463.13223

**Published:** 2021-09-30

**Authors:** Yue Bin, Yanyao Liu, Shaoqiu Jiang, Hui Peng

**Affiliations:** ^1^ Department of Ophthalmology Chongqing Key Laboratory of Ophthalmology and Chongqing Eye Institute The First Affiliated Hospital of Chongqing Medical University Chongqing China; ^2^ Department of Ophthalmology The Second Affiliated Hospital of Chongqing Medical University Chongqing China

**Keywords:** age‐related macular degeneration, choroidal neovascularization, YKL‐40, vascular endothelial growth factor

## Abstract

Choroidal neovascularization (CNV) is a key characteristic of wet age‐related macular degeneration (AMD) that can lead to severe vision loss in the elderly. Anti‐VEGF therapy is currently the premier strategy for wet AMD, but it has limited efficacy. Previous studies have shown that chitinase‐3‐like‐1 (YKL‐40) can promote microangiogenesis and inflammation, but its effect on CNV formation has not yet been studied. Here, we investigated the potential role of YKL‐40 in wet AMD and the underlying mechanism(s). We report that the serum expression of YKL‐40 in wet AMD patients was significantly higher than that in control patients and was positively correlated with VEGF expression, indicating that YKL‐40 may participate in the development of wet AMD. In addition, YKL‐40 and VEGF expression levels were observed to be increased and the ERK1/2 pathway activated in the neuroretinal (NR) and RPE/choroid tissues of mice with laser‐induced CNV. The YKL‐40 and phosphorylated protein levels of the ERK1/2 pathway were decreased after intravitreal injection with an anti‐YKL‐40 antibody, suggesting that anti‐YKL‐40 could inhibit the activation of the ERK1/2 pathway. These results indicate that YKL‐40 may serve as a novel target for the diagnosis and treatment of wet AMD.

## Graphical abstract

We demonstrate that serum expression of YKL‐40 in wet AMD patients was significantly higher than that in control patients. YKL‐40 and phosphorylated protein levels of the ERK1/2 pathway in mice with laser induction were decreased after intravitreal injection with an anti‐YKL‐40 antibody, indicating that YKL‐40 may serve as a novel target for the diagnosis and treatment of wet AMD.

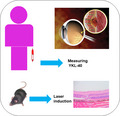

**Abbreviations**
AMD: age‐related macular degeneration
CNV: choroidal neovascularization
ERK1/2: extracellular regulated protein kinases
RPE: retinal pigment epithelium
VEGF: vascular endothelial growth factor
YKL‐40: Chitinase‐3‐like‐1

Age‐related macular degeneration (AMD) is the leading cause of irreversible vision impairment in aging populations [1, 2, 3]. Choroidal neovascularization (CNV), a typical characteristic of wet AMD, is the main reason for vision loss. Although the exact mechanism underlying CNV development has not been fully elucidated, vascular endothelial growth factor (VEGF) plays a vital role in the process of CNV [4], and anti‐VEGF agents can be used to ameliorate the clinical outcomes of wet AMD patients. However, only 30–40% of patients have enhanced visual acuity after treatment with intravitreal injection of anti‐VEGF [5], which suggests that in addition to VEGF, there may be other mechanisms involved in the formation of CNV.

The chitinase‐3‐like‐1 (CHI3L1) protein, also called YKL‐40, a mammalian member of the 18‐glycosyl hydrolase family [6, 7], can be generated and secreted by various cells, such as macrophages, neutrophils, and vascular smooth muscle cells [8]. YKL‐40 is involved in cell proliferation, microangiogenesis, and inflammation. Elevated serum YKL‐40 was observed in multiple diseases, including rheumatoid arthritis, osteoarthritis, giant cell arteritis, pneumonia, and diverse tumors [9]. Rakic reported that YKL‐40 was not only expressed in the normal human retina and RPE but also highly expressed in the CNV tissues of patients; the author also reported that estrogen could reduce YKL‐40 expression in mice [10]. Increased YKL‐40 in many types of tumors is able to facilitate angiogenesis by motivating the migration and rearrangement of vascular endothelial cells [11], while an anti‐YKL‐40‐neutralizing antibody has been identified to have an inhibitory effect on tumor growth and neovascularization [12, 13, 14]. However, to our knowledge, the expression and effect of YKL‐40 in wet AMD have not yet been clarified.

In this study, we analyzed the correlation between YKL‐40 and VEGF, which was previously reported to be closely related to wet AMD. As neovascularization is the hallmark of wet AMD, we aimed to verify whether the serum concentrations of YKL‐40 in patients with wet AMD were elevated and the diagnostic accuracy of serum YKL‐40 to predict wet AMD. Additionally, we investigated the possible mechanism of YKL‐40 in mice with laser‐induced CNV formation.

## Methods

### Study subjects

Subjects from the First Affiliated Hospital of Chongqing Medical University were enrolled. AMD was diagnosed by detailed medical history, slit‐lamp microscopy, fundus photography, optical coherence tomography (OCT), and fluorescein fundus angiography (FFA). The inclusion criteria for male and female participants of both genders were as follows: ≥ 50 years old, nondiabetic, nonhypertensive, and no other ocular complication(s) except AMD. Subjects suffering from arthritis, cancer, and/or use of anti‐inflammatory or anti‐VEGF therapeutics were excluded from the study. Control subjects were required to meet the same inclusion criteria and underwent detailed examinations for confirmation of healthy ocular conditions. Consequently, 65 subjects who fulfilled the inclusion criteria were divided into the control (*n* = 20) and AMD (*n* = 45) groups. Clinical parameters of all the subjects such as gender, age, smoking history, low‐density lipoprotein cholesterol (LDL‐C), high‐density lipoprotein cholesterol (HDL‐C), total cholesterol (TC), and high sensitivity C‐reactive protein (hs‐CRP) were recorded. All participants signed written informed consent, and the study protocol was approved by the Research Ethics Committee of the First Affiliated Hospital of Chongqing Medical University. All procedures were conducted in accordance with the Helsinki declaration.

### Sample acquisition and storage

Peripheral venous blood samples (5 mL) were gathered and centrifuged at 3000 × ***g*** for 5 min, immediately aliquoted, and stored at −80 °C until further measurement.

### ELISA

The serum concentrations of YKL‐40 and VEGF were quantified by corresponding ELISA kits (#DC3L10, #DVE00; R&D Systems, USA) according to the protocols. The absorbance at the wavelength of 450 nm was determined with the use of microplate reader (Varioskan + LUX; Thermo Fisher, USA) in the dark.

### Animals

A total of 60 male C57BL/6J mice aged 6 to 8 weeks were obtained from the Laboratory Animal Center of Chongqing Medical University and raised in the center under a 12‐/12‐h light‐dark cycle. All procedures involving animals were approved by the Ethics Committee of the First Affiliated Hospital of Chongqing Medical University.

### Laser‐induced CNV and intravitreal injection in mice

After undergoing anesthesia with pentobarbital sodium (1% w/v) and pupil dilation with tropicamide, the mice were subjected to laser‐induced photocoagulation (532 nm, 0.1 s, 100 μm, 150 mW) by a krypton‐argon laser (Novus Varia Dpss; Lumenis, USA). Five laser spots were made in each eye to induce CNV, with white bubbles indicating Bruch’s membrane rupture at each spot. After laser injury, intravitreal injection was performed immediately through a 35‐gauge needle attached to a 10 μL nanofilm syringe. Then, 2 μL of neutralizing monoclonal anti‐YKL‐40 antibody (Yemai Biotechnology, China) or PBS solution was gently inserted into the vitreous cavity.

### Hematoxylin–eosin staining of paraffin sections

After mice were euthanized, the eyeballs were dissected and fixed with 4% formaldehyde solution for 24 h, followed by dehydration with a sucrose gradient before they were embedded in paraffin wax. Sagittal sections at the CNV lesion were selected, and 6.0‐μm‐thick slices were prepared for staining with hematoxylin‐eosin (HE) before they were photographed under a light microscope.

### Real‐time PCR analysis

YKL‐40 and VEGF mRNA expression at 7 and 14 days after laser induction was examined by real‐time PCR. After the mice were sacrificed, the eyeballs were dissected using an anatomical microscope, and the peripheral fascia of the mice was cut off. The eyeballs were cut along the limbus of the cornea, and retinal tissue could be observed behind the crystal and vitreous separation. The neuroretina (NR) and retinal pigment epithelium (RPE)/choroid were dissected from the eyeballs of the mice [15]. These two tissues were separately processed for total RNA using TRIzol (Takara Biotechnology, China), and reverse transcription was subsequently performed to synthesize complementary DNA (cDNA) using the Prime Script RT reagent kit (Takara Biotechnology, China). Real‐time PCR (SYBR Green) was carried out in the ABI 7500 system (Applied Biosystems, USA). The PCR primers were as follows: 5′‐ACCAACCTGAAGACCCTCCT‐3′ and 5′‐GCCCATCAAAGCCATAAGAA‐3′ for YKL‐40; 5′‐TCACCACCACGCCATCATC‐3′ and 5′‐AATGGCGAATCCAGTCCCA‐3′ for VEGF; and 5′‐GTATGACTCCACTCACGGCAAA‐3′ and 5′‐ GGTCTCGCTCCTGGAAGATG‐3′ for GAPDH.

### Western blot analysis

On days 7 and 14 after laser induction, NR and RPE/choroid tissues were dissected from mice, homogenized, and lysed in RIPA buffer mixed with 1% protease inhibitor (Beyotime, China). After centrifugation of the lysates at 15 984 ***g*** for 15 min, the supernatants were gathered and diluted in loading buffer (Beyotime, China). The proteins were separated by SDS/PAGE and electrophoretically transferred to PVDF membranes (Millipore, Billerica, MA, USA). After being blocked, primary antibodies against YKL‐40 (#47066; Cell Signaling Technology, USA), VEGF (ab69479; Abcam, UK), ERK1/2 (#4695; Cell Signaling Technology), p‐ERK1/2 (#4370; Cell Signaling Technology, USA), and GAPDH (#2118; Cell Signaling Technology) were used to probe protein. The membranes were incubated with secondary antibodies and analyzed by enhanced chemiluminescence (ECL; Millipore).

### Statistical analysis

Data are presented as the means ± SEM and analyzed using spss Statistics 20.0 software (IBM, NY, USA). Student’s *t* test was used to compare serum levels of YKL‐40 and VEGF. The Pearson correlation coefficient was computed for YKL‐40 and VEGF expression levels. The predictive capabilities (i.e., diagnostic performances) of YKL‐40 and VEGF were investigated by construction of the area under the receiver operating characteristic (ROC) curve (AUC). One‐way ANOVA with Bonferroni correction was used to analyze comparisons of three groups. *P* values less than 0.05 were considered statistically significant.

## Results

### Baseline characteristics

The study population comprised 45 patients (female : male, 2 : 3) and 20 healthy controls (female : male, 1 : 2). The AMD patients were slightly older than the control patients, with average ages of 71.36 ± 10.19 years and 69.65 ± 9.06 years, respectively. No significant difference was observed in age nor sex between the two groups. Moreover, no significant differences were found in LDL‐C, HDL‐C, TC, hs‐CRP, and smoking between the AMD group and the control group (Table 1).

**Table 1:** Comparison between AMD patients and healthy controls: general information and biochemical data. LDL‐C, low‐density lipoprotein cholesterol; HDL‐C, high‐density lipoprotein cholesterol; TC, total cholesterol; hs‐CRP, high sensitivity C‐reactive protein.

	Control group	AMD group	*P* value
*N*, total	20	45	
Male (%)	66.67	60.00	0.6050
Age (years)	69.65 ± 9.06	71.36 ± 10.19	0.5233
Smoking (%)	40.00	37.78	0.8650
LDL‐C (mmol·L^−1^)	2.75 ± 0.07	2.78 ± 0.04	0.7204
HDL‐C (mmol·L^−1^)	1.57 ± 0.06	1.48 ± 0.04	0.1964
TC (mmol·L^−1^)	4.14 ± 0.35	4.46 ± 0.20	0.4049
Hs‐CRP (mg·L^−1^)	2.45 ± 0.52	2.66 ± 0.54	0.8161

### Serum levels of YKL‐40 and VEGF in AMD patients and healthy controls

**Fig. 1:**
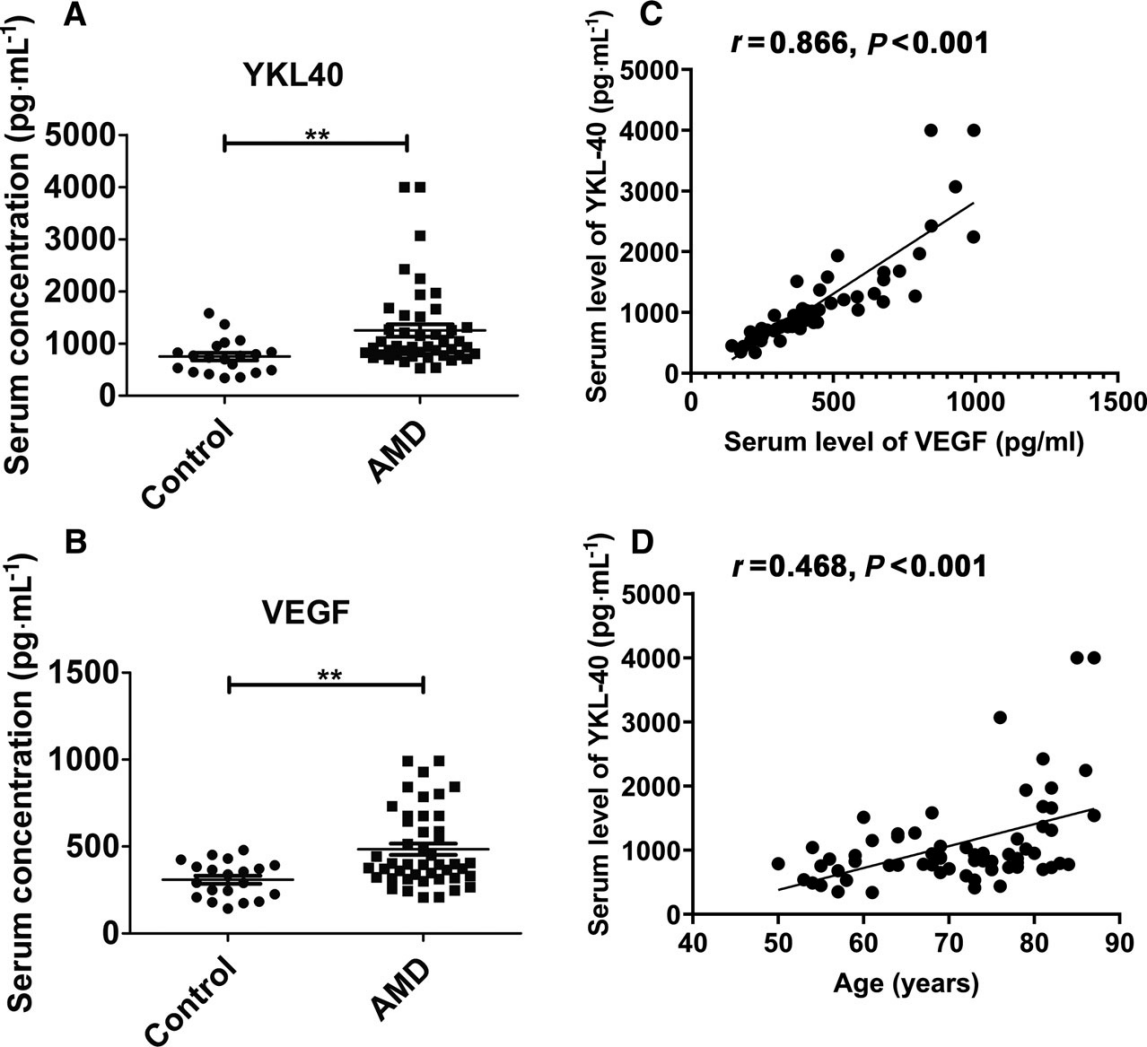
The serum expression of YKL‐40 and VEGF in patients with AMD. (A) The serum expression of YKL‐40 in 45 patients with wet AMD and 20 healthy controls (control). (B) Serum VEGF in 45 patients with wet AMD and 20 healthy controls. (C) The correlation between serum YKL‐40 and VEGF in the participants. (D) The correlation between serum YKL‐40 levels and patient age. Student’s t test was used to compare serum levels of YKL‐40 and VEGF. The association between YKL‐40 and either VEGF or age was investigated with the Pearson correlation. *P* < 0.05 denotes statistical significance, **P < 0.01.

In the wet AMD group, the serum concentration of YKL‐40 (1251.84 ± 118.94 pg·mL^−1^) was markedly higher than that in the healthy subjects (751.19 ± 73.79 pg·mL^−1^, *P* = 0.0090) (Fig. 1A). The same trend was observed in regard to VEGF levels, as the VEGF concentration in the patients with wet AMD (484.19 ± 32.57 pg·mL^−1^) was obviously higher than that in the healthy participants (309.35 ± 23.15 pg·mL^−1^, *P* = 0.0018) (Fig. 1B).

### Correlation between serum YKL‐40 and VEGF

Pearson's correlation analysis illustrated a positive correlation between serum YKL‐40 and VEGF (Pearson's *r* = 0.866, *P* < 0.001) (Fig. 1C). Additionally, a positive correlation was also observed between YKL‐40 and age (Pearson's *r* = 0.468, *P* < 0.001) (Fig. 1D).

### Diagnostic value of serum YKL‐40 and VEGF for wet AMD

**Fig. 2:**
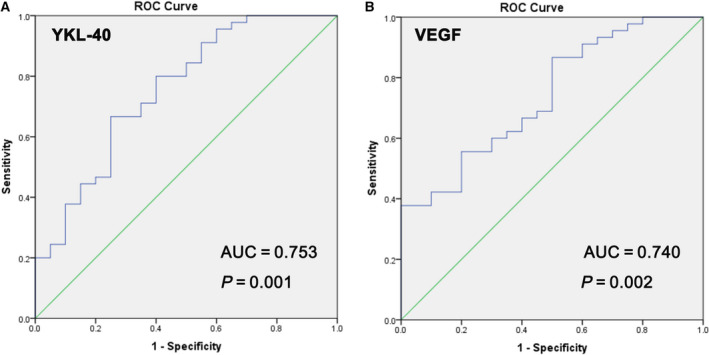
ROC curves for quantitative serum expression of YKL‐40 and VEGF in the AMD group compared with the control group. In the illustration, AUC values homologous to the area under the ROC curve are reported. (A) ROC curves for serum YKL‐40. (B) ROC curves for serum VEGF.

To test the diagnostic accuracies of YKL‐40 and VEGF for wet AMD, ROC curve analyses were performed. As demonstrated in Fig. 2A, serum YKL‐40 may be regarded as a significant predictor of wet AMD. The AUC for YKL‐40 was 0.753 (95% CI 0.622–0.885) at a cutoff value of 835.95 pg·mL^−1^, with 66.70% sensitivity and 75.00% specificity (*P* = 0.001). Similarly, VEGF had an area under the ROC curve of 0.740 (95% CI 0.614–0.866) and a cutoff value of 393.66 pg·mL^−1^, with 55.60% sensitivity and 80.00% specificity (*P* = 0.002) (Fig. 2B).

### Increased mRNA expression of YKL‐40 and VEGF in mice with laser‐induced CNV

**Fig. 3:**
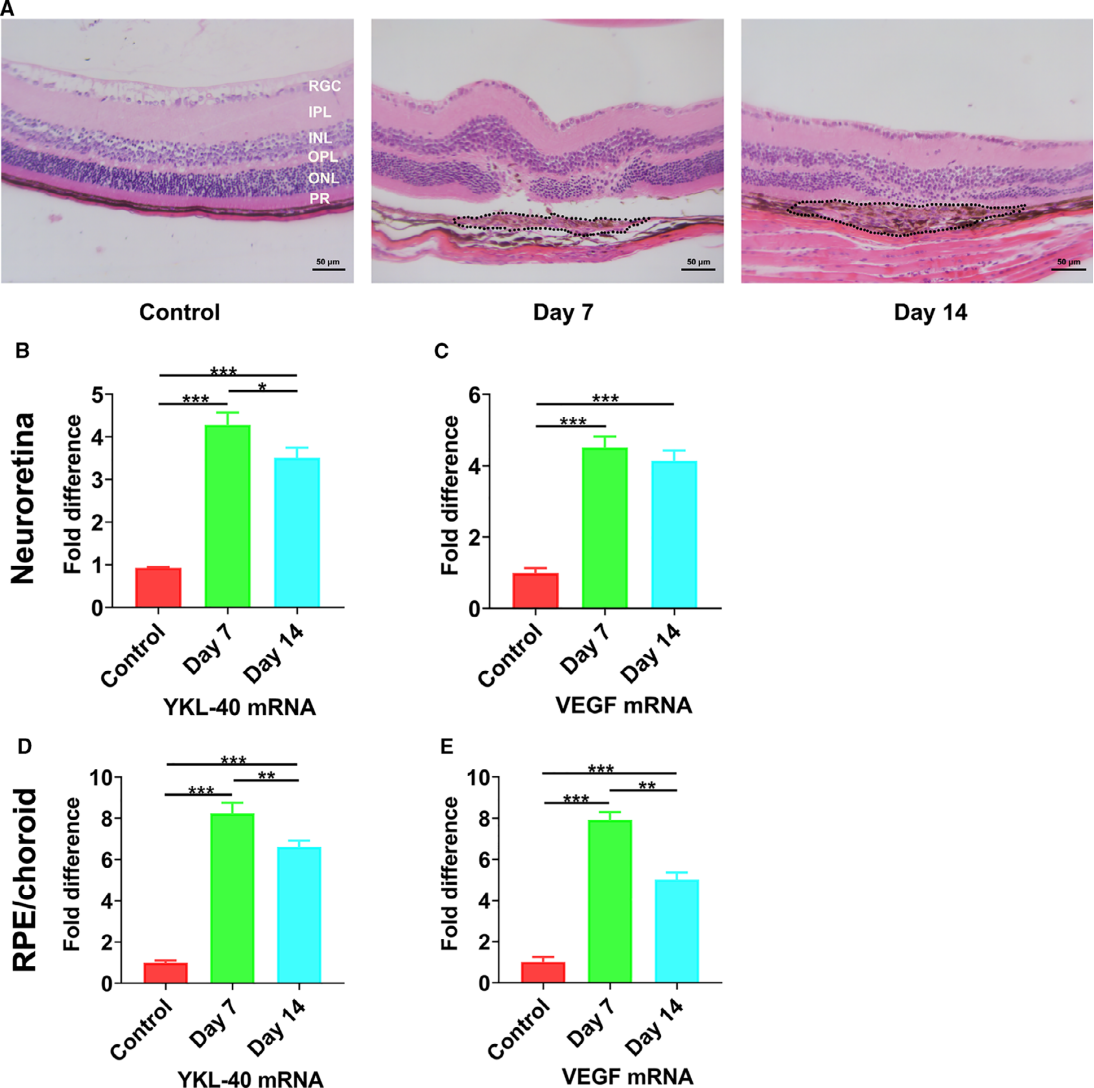
Detection of CNV in mice by HE staining and of YKL‐40 and VEGF mRNA levels. (A) The HE staining results showed normal mouse retinal and choroid structures and the areas of CNV at 7 and 14 days postlaser photocoagulation (RGC, retinal ganglion cell layer; IPL, inner plexiform layer; INL, inner nuclear layer; OPL, outer plexiform layer; ONL, outer nuclear layer; PR: photoreceptor). Scale bar = 50 μm. (B–E) Analysis of YKL‐40 and VEGF mRNA expression in the NR and RPE/choroid of mice with laser‐induced CNV. (Mean ± SEM; *n* = 4; one‐way ANOVA with Bonferroni correction). *P* < 0.05 denotes statistical significance, *P < 0.05, **P < 0.01, ***P < 0.001.

We used laser photocoagulation to establish a CNV mouse model and then determined whether YKL‐40 and VEGF expression was elevated in the NR and RPE/choroid tissues. Histological analysis of retina stained with HE confirmed CNV formation on days 7 and 14 after the ruptured Bruch′s membrane induced by laser, with a disordered retinal structure, abnormal cell proliferation, and loss of the outer nuclear layer at the laser‐damaged area (Fig. 3A).

To investigate the mRNA expression of YKL‐40 and VEGF, real‐time PCR was performed. Compared with the control group, the mRNA levels of YKL‐40 in both the Day 7 and Day 14 groups were markedly increased in the neuroretina (*P < 0.05) (Fig. 3B). Consistent with the YKL‐40 expression, the expression of VEGF in the neuroretina was also significantly upregulated after laser induction on days 7 and 14 (***P < 0.001) (Fig. 3C). Furthermore, YKL‐40 and VEGF mRNA expression in the RPE/choroid complex was significantly increased in both the Day 7 and Day 14 groups (**P < 0.01) (Fig. 3D and E).

### Elevated protein expression of YKL‐40 and VEGF and activation of the ERK1/2 pathway after laser induction

**Fig. 4:**
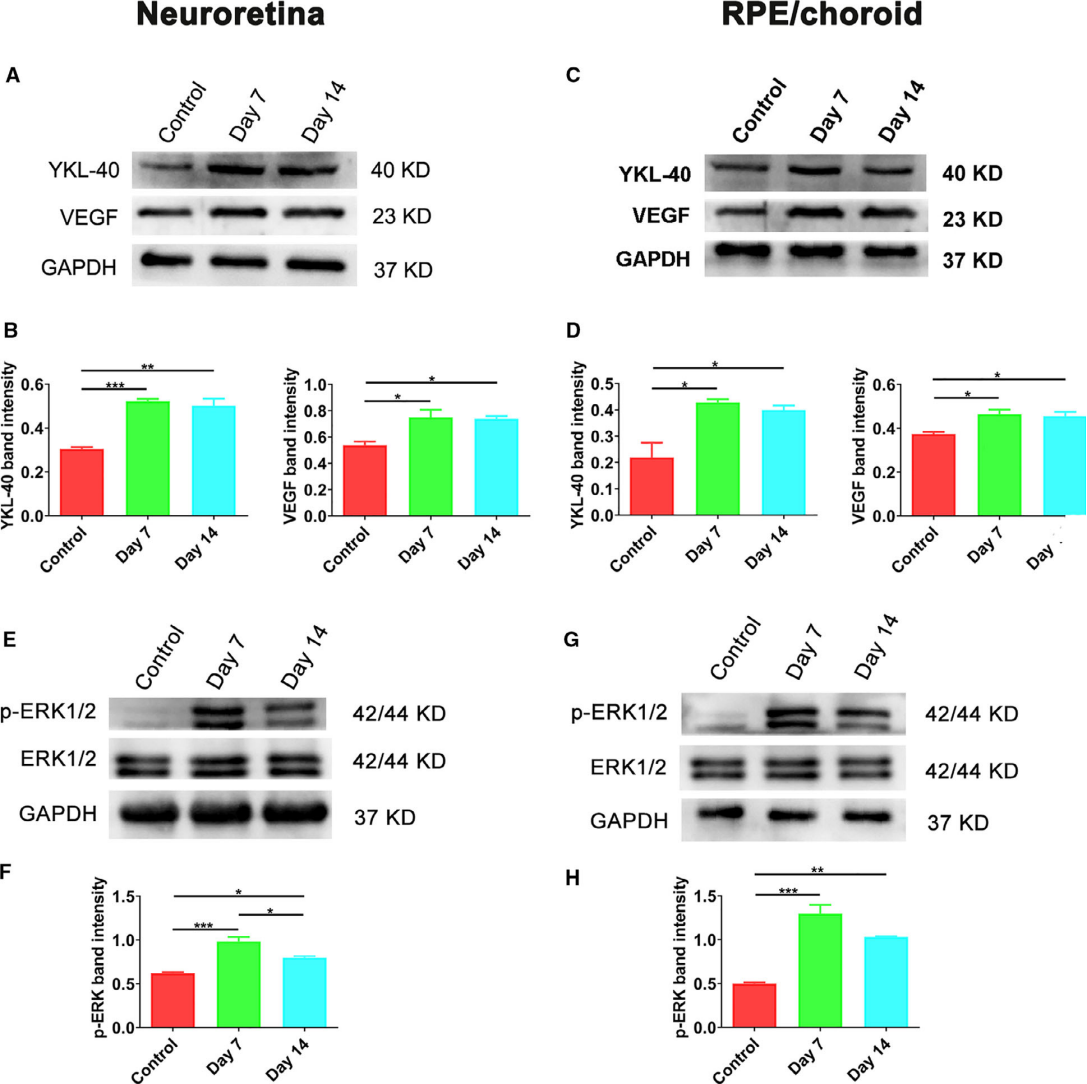
YKL‐40 and VEGF protein expression and ERK1/2 signaling are upregulated in mice with laser‐induced CNV. (A–B) The protein levels of YKL‐40 and VEGF in the neuroretinas of mice after laser photocoagulation. (C–D) The protein levels of YKL‐40 and VEGF in the RPE/choroid complex of mice after laser photocoagulation. (E–H) ERK1/2 phosphorylation levels were upregulated in the NR and RPE/choroid of mice subjected to laser photocoagulation. (Mean ± SEM; *n* = 4; one‐way ANOVA with Bonferroni correction). *P* < 0.05 denotes statistical significance, *P < 0.05, **P < 0.01, ***P < 0.001.

The western blot results showed that the protein expression levels of YKL‐40 and VEGF in both the NR and RPE/choroid tissues were significantly higher in the Day 7 and Day 14 groups after laser photocoagulation than those the control group (*P < 0.05) (Fig. 4A–D). To further determine whether the ERK1/2 pathway is involved in laser‐induced CNV formation, the levels of phosphorylated ERK1/2 in the NR and RPE/choroid were examined 7 and 14 days after laser induction. The phosphorylation levels of ERK1/2 in the two tissues were remarkably increased in the Day 7 and Day 14 groups compared to the control group (*P < 0.05) (Fig. 4E–H).

### Decreased CNV area, YKL‐40, and phosphorylated ERK1/2 pathway protein levels after intravitreal injection of anti‐YKL‐40 antibody

**Fig. 5:**
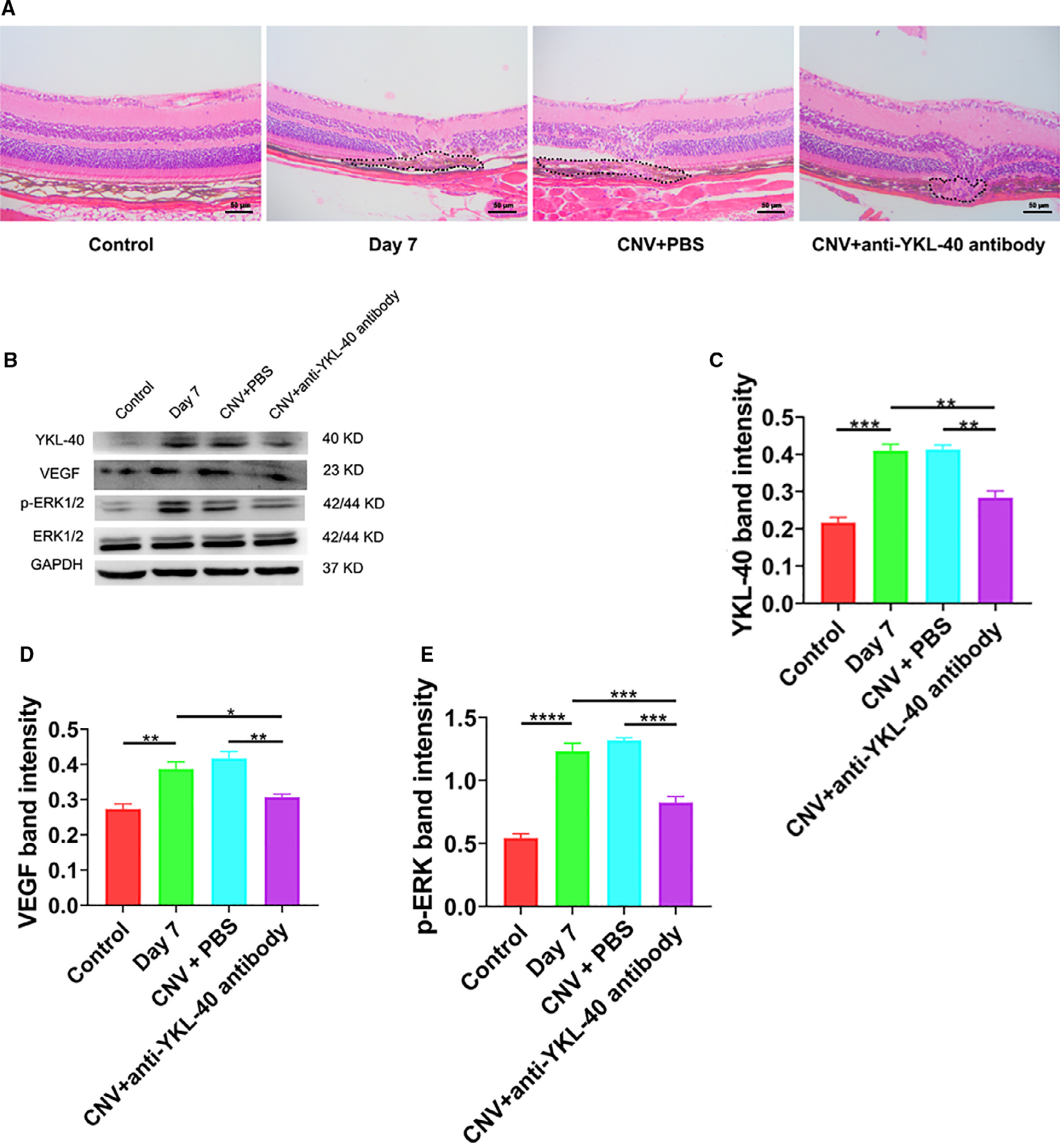
CNV area, YKL‐40, and phosphorylated protein level of ERK1/2 pathway are decreased after intravitreal injection of the anti‐YKL‐40 antibody. (A) The HE staining results showed mouse retina structures and the areas of CNV after intravitreal injection of the anti‐YKL‐40 antibody or PBS solution postlaser photocoagulation. Scale bar = 50 μm. (B–E) The protein levels of YKL‐40, VEGF, and phosphorylated ERK1/2 in the RPE/choroid complex of the mice after intravitreal injection of anti‐YKL‐40 antibody or PBS solution postlaser photocoagulation. (Mean ± SEM; *n* = 4; one‐way ANOVA with Bonferroni correction). *P* < 0.05 denotes statistical significance, *P < 0.05, **P < 0.01, ***P < 0.001, ****P < 0.0001.

The CNV area was observed on the 7th day after intravitreal injection of the anti‐YKL‐40 antibody. Compared with that of the PBS group and the Day 7 group, the CNV area was reduced (Fig. 5A). The protein expression levels of YKL‐40 and phosphorylated ERK1/2 in RPE/choroid tissues were significantly lower after intravitreal injection of the anti‐YKL‐40 antibody than those of the CNV group (*P < 0.05) (Fig. 5B–E).

## Discussion

YKL‐40 is classified into the chitinase‐like protein (CLP) family, the members of which do not provide enzymatic activity to degrade chitin directly. The association with the biological activity of YKL‐40 and the regulation of cell proliferation, activation, migration, and adhesion was previously investigated [16]. Although increased serum YKL‐40 and VEGF in many diseases have been demonstrated [17, 18, 19, 20, 21], very few studies have investigated YKL‐40, especially with regard to the correlation of YKL‐40 and VEGF in patients with wet AMD.

Herein, serum YKL‐40 levels were significantly elevated in patients with wet AMD and positively correlated with VEGF expression, implying the involvement of YKL‐40 in the pathological process of wet AMD. Additionally, moderate sensitivity and specificity values (66.70% and 75%, respectively, *P* = 0.001) were found when a cutoff level of 835.95 pg·mL^−1^ was used, which suggested that YKL‐40 could be considered a predictor of wet AMD. Currently, several studies have demonstrated increased serum YKL‐40 in patients with inflammatory diseases and different types of solid tumors, indicating the important role of YKL‐40 in inflammation and tumor development. Moreover, YKL‐40 is deemed a new biomarker used to estimate disease activity in inflammation. Studies have reported elevated serum YKL‐40 and a positive correlation of serum YKL‐40 and disease activity in rheumatoid arthritis [22]. Numerous studies have revealed that elevated serum YKL‐40 is associated with disease severity, which may correspond to poor prognosis in many diseases, including cancer, type 2 diabetes, and coronary artery disease [23, 24, 25].

Furthermore, in line with the human serum results, both the mRNA and protein expression levels of YKL‐40 and VEGF were significantly increased in mice after laser induction compared to control mice. These results are consistent with a published report describing that YKL‐40 expression was upregulated in both pathological human and experimental CNV samples [10], which may suggest a link between YKL‐40 and CNV formation in wet AMD. Extensive *in vivo* and *in vitro* studies have demonstrated that YKL‐40 plays a potential role in cancer‐related angiogenesis by promoting endothelial cell migration and participating in vascular structure formation [26, 27]. *In vitro* studies using human microvascular endothelial cells (HMVECs) and the U87 human glioma cell line showed that YKL‐40 and VEGF had a synergistic effect on vascular structure formation [26, 27]. *In vivo* experiments involving mouse models of human breast cancer, colon cancer and glioma as well as material from patients with glioma highlighted the role of YKL‐40 in promoting angiogenesis [28]. In addition, a positive correlation was shown between YKL‐40 and angiogenesis markers such as CD31, CD34, and VEGFD in invasive ductal breast carcinoma, which also suggests a link between YKL‐40 and angiogenesis [29].

MAPK, a common intracellular pathway, plays a crucial role in carcinogenesis, promoting cancer cell proliferation and migration, inhibiting apoptosis and stimulating angiogenesis [30, 31, 32]. Under normal circumstances, ERK1/2 is located in the cytoplasm. It is phosphorylated and activated after being stimulated by high glucose, various growth factors, hydrogen peroxide, ion rays, etc., promoting gene transcription and expression, and participating in cell proliferation and differentiation. In mammalian cells, the intracellular signal transduction pathway associated with ERK is considered to be the classic MAPK signal transduction pathway. In the present study, we demonstrated that ERK1/2 pathway was activated after laser induction. Moreover, the YKL‐40 and phosphorylated protein levels of the ERK1/2 pathway were decreased after intravitreal injection of the anti‐YKL‐40 antibody, suggesting that anti‐YKL‐40 could inhibit the activation of the ERK1/2 pathway. Recklies *et al*. [33] showed that YKL‐40 is a strong inductor of MAPK signal pathway, mediating its action through the phosphorylation of ERK1/2. More than our results, a majority of *in vitro* studies have reported that YKL‐40 participates in angiogenesis by activating the MAPK/ERK pathway [8, 27, 34]. Although YKL‐40 can induce syndecan‐1 (SDC1) to bind to different integrins in different cells, thereby activating different kinases, neovascularization is the ultimate goal. In endothelial cells, YKL‐40 promotes the formation of the bond between SDC1 and integrin αvβ3, which leads to activation of the angiogenesis‐promoting ERK1/2 pathway through focal adhesion kinase (FAK861) [8]. In glioma cells, YKL‐40 promotes the formation of the bond between SDC1 and αvβ5 and further activates the ERK1/2 pathway through FAK397, thereby increasing the expression of VEGF and promoting angiogenesis [27]. Unfortunately, how YKL‐40 activates the ERK pathway and induces CNV in wet AMD has not yet been discovered. Therefore, it needs to be further studied.

In this research, we utilized a single‐center clinical study with a small sample size and inevitable bias. In future studies, we plan to expand the sample size and consider multicenter cooperation, extend patient follow‐up time, compare serum YKL‐40 levels before and after anti‐VEGF treatment, and provide more reliable evidence for YKL‐40 in the diagnosis and treatment of wet AMD.

## Conclusions

In conclusion, compared with healthy controls, patients with wet AMD exhibited a higher serum YKL‐40 expression and a positive correlation between YKL‐40 and VEGF expression, which indicates the possible involvement of YKL‐40 in wet AMD. The YKL‐40 and phosphorylated protein levels of the ERK1/2 pathway were decreased after intravitreal injection of the anti‐YKL‐40 antibody, suggesting that anti‐YKL‐40 could inhibit the activation of the ERK1/2 pathway. These results are a prelude to further studying the functional and in‐depth mechanisms of YKL‐40 in wet AMD.

## Conflict of interest

The authors declare no conflict of interest.

## Author contributions

HP conceived and designed the study. YB, YL, and SJ. carried out the experiments. YB and YL analyzed the data and edited the figure. YB wrote the article. HP revised the article. All authors read and agreed on the final version of the article.

## Data Availability

The analyzed datasets generated during the study are available from the corresponding author on reasonable request.
